# Ets Family Transcription Factor Fli-1 Promotes Leukocyte Recruitment and Production of IL-17A in the MRL/Lpr Mouse Model of Lupus Nephritis

**DOI:** 10.3390/cells9030714

**Published:** 2020-03-14

**Authors:** Shuzo Sato, Xian K. Zhang, Jumpei Temmoku, Yuya Fujita, Naoki Matsuoka, Makiko Yashiro-Furuya, Tomoyuki Asano, Hiroko Kobayashi, Hiroshi Watanabe, Kiyoshi Migita

**Affiliations:** 1 Department of Rheumatology, Fukushima Medical University School of Medicine, Fukushima 960-1295, Japan; temmoku@fmu.ac.jp (J.T.); fujita31@fmu.ac.jp (Y.F.); naoki-11@fmu.ac.jp (N.M.); myashiro@fmu.ac.jp (M.Y.-F.); asanovic@fmu.ac.jp (T.A.); hkoba@fmu.ac.jp (H.K.); chiehiro@fmu.ac.jp (H.W.); migita@fmu.ac.jp (K.M.); 2 Division of Rheumatology and Immunology, Medical University of South Carolina, Charleston, SC 29425, USA

**Keywords:** Fli-1, MRL/lpr mouse, lupus nephritis, IL-17A, interstitial inflammation, CCL20, chemokines

## Abstract

The transcription factor Friend leukemia integration 1 (Fli-1) regulates the expression of numerous cytokines and chemokines and alters the progression of lupus nephritis in humans and in the MRL/MpJ-*Fas^lpr^* (MRL/lpr) mouse model. Th17-mediated immune responses are notably important as they promote ongoing inflammation. The purpose of this study is to determine the impact of Fli-1 on expression of interleukin-17A (IL-17A) and the infiltration of immune cells into the kidney. IL-17A concentrations were measured by ELISA in sera collected from MRL/lpr *Fli-1*-heterozygotes (*Fli-1^+/−^*) and MRL/lpr *Fli-1^+/+^* control littermates. Expression of IL-17A and related proinflammatory mediators was measured by real-time polymerase chain reaction (RT-PCR). Immunofluorescence staining was performed on renal tissue from MRL/lpr *Fli-1^+/^^−^* and control littermates using anti-CD3, anti-CD4, and anti-IL-17A antibodies to detect Th17 cells and anti-CCL20 and anti-CD11b antibodies to identify CCL20^+^ monocytes. The expression of IL-17A in renal tissue was significantly reduced; this was accompanied by decreases in expression of IL-6, signal transducer and activator of transcription 3 (STAT3), and IL-1β. Likewise, we detected fewer CD3^+^IL-17^+^ and CD4^+^IL-17^+^ cells in renal tissue of MLR/lpr *Fli-1^+/^^−^* mice and significantly fewer CCL20^+^CD11b^+^ monocytes. In conclusion, partial deletion of Fli-1 has a profound impact on IL-17A expression and on renal histopathology in the MRL/lpr mouse.

## 1. Introduction

Systemic lupus erythematosus (SLE) is a chronic autoimmune disease of unknown etiology that is associated with chronic inflammation in multiple organs including the kidney [1]. Infiltration of inflammatory cells into the kidney, a condition known as lupus nephritis, can ultimately result in renal failure [2]. Friend leukemia integration 1 (Fli-1) is a member of the Ets family of transcription factors and binds to DNA sequences that include a consensus GGA(A/T) motif. Fli-1 can function as either a transcriptional activator or repressor and has been detected in endothelial cells, fibroblasts, and several hematopoietic lineages [3,4,5,6]. We previously reported that Fli-1 plays an important role in the development of lupus nephritis [7,8,9,10,11,12,13,14,15]. While overexpression of Fli-1 results in lupus-like glomerulonephritis [9], homozygous Fli-1 gene deletion (Fli-1*^−^*^/*−*^) results in fetal demise in association with neural tube hemorrhage [4]. Nonetheless, we found that the heterozygous state (Fli-1^+/*−*^) yielded viable progeny that experienced reduced renal inflammation and prolonged survival in the MRL/lpr mouse lupus model [7,8]. Our previous studies demonstrated that Fli-1 regulates cytokine and chemokine expression in MRL/lpr mice and modulates production of critical mediators associated with lupus nephritis, including monocyte chemoattractant protein-1 (MCP-1), Regulated on Activation, Normal T Cell Expressed and Secreted (RANTES), granulocyte colony stimulating factor (G-CSF), chemokine (C-X-C motif) ligand 2 (CXCL2), and interleukin-6 (IL-6) [10,11,12,13,14,15]. Likewise, kidneys from *Fli-1^+/−^* lupus-prone NZM2410 mice displayed significantly fewer inflammatory infiltrates compared to the *Fli-1^+/+^* NZM2410 controls [8]. Interestingly, peripheral blood lymphocytes from patients with active SLE showed elevated expression of Fli-1 transcripts in parallel with disease activity [16]; elevated levels of Fli-1 were also associated with newly-developed or recurrent lupus nephritis in SLE patients [17]. The cytokine IL-17A plays an important role in the development of lupus nephritis; it is produced by T-helper 17 (Th17) cells and has powerful inflammatory properties [18,19]. Previous reports indicated that IL-17A is associated with disease activity in patients with SLE as well as with lupus nephritis [20,21,22,23]. Not only CD4^+^ T cells, but also CD3^+^CD4*^−^*CD8*^−^* double-negative T cells were reported to be major producers of IL-17A in the kidney of lupus nephritis patients [24,25]. In this report, we investigate the impact of Fli-1 and its role in the development of lupus nephritis. To do so, we examine the impact of Fli-1 on IL-17A expression in the kidney and its role in promoting cellular infiltration in the MRL/lpr mouse model.

## 2. Materials and Methods

### 2.1. Mice

Wild-type (WT) MRL/lpr *Fli-1^+/+^* mice and MRL/lpr *Fli-1^+/−^* mice were generated as previously reported [7]. MRL/lpr *Fli-1^+/−^* mice were generated by back-crossing with *Fli-1^+/−^* C57BL/6 strain for more than 12 generations, as described in our previous report [7]. All mice were housed under pathogen-free conditions in the animal institute of Fukushima Medical University. All animal experiments were approved by the institutional review board at Fukushima Medical University (No. 300068). This study was conducted in accordance with the Declaration of Helsinki.

### 2.2. Genotyping by Polymerase Chain Reaction (PCR)

Mice were genotyped using PCR to differentiate between the wild-type and mutant Fli-1 alleles as reported previously [7]. Briefly, the PCR primers used include: Fli-1 exon IX/forward primer (positions 1156–1180), 5′-GACCAACGGGGAGTTCAAAATGACG-3′; Fli-1 exon IX/reverse primer (positions 1441–1465), 5′-GGAGGATGGGTGAGACGGGACAAAG-3′; and Pol II/reverse primer, 5′-GGAAGTAGCCGTTATTAGTGGAGAGG-3′. DNA was isolated from tail snips of 4-week old mice using the QIAamp Tissue Kit (QIAGEN). PCR analyses were performed using C1000 Touch Thermal Cycler (Bio-Rad).

### 2.3. Histopathology, Immunohistochemistry, and Immunofluorescence Staining

Kidneys were removed from adult mice, fixed in formalin, and embedded in paraffin. The deparaffinized section was stained with hematoxylin and eosin (H&E). Kidney sections were evaluated by light microscopy and the grade of kidney inflammation was evaluated using the pathology scoring system that was previously described [7]. Briefly, H&E stained kidney sections were evaluated in a blinded fashion and graded for glomerular inflammation, proliferation, crescent formation, and necrosis. Scores from 0 to 3 (0, none; 1, mild; 2, moderate; and 3, severe) were applied for each of these features and then summed together to yield a final pathology score. Scoring of interstitial inflammation was as follows: score 0, <5% involvement; score 1, 5–25% involvement; score 2, 25–50% involvement; and score 3, >50% involvement [2]. IL-17A was detected in tissues with rabbit polyclonal anti-IL-17A antibodies (Abcam) and the standard ultra-sensitive avidin-biotin complex (ABC) method (Vector). CD3^+^, CD4^+^, and IL-17A^+^ cells were detected in tissue with FITC-conjugated rat anti-mouse CD3 or anti-mouse CD4 antibodies (Biolegend) and rabbit polyclonal anti-IL-17A (Abcam) with secondary anti-rabbit IgG conjugated to Alexa Fluor^®^ 647 (Abcam). Similarly, FITC-conjugated anti-mouse CD11b (Biolegend) and anti-CCL20 (Abcam) were used to detect immunoreactive CD11b cells and CCL20 (a representative chemoattractant for Th17 cells), respectively [26]. Detection of CD3^+^, CD4^+^, and CD11b^+^ cells within the kidney infiltrates (mainly interstitial inflammation) was as described previously [2,14]. Briefly, 10 random sites were counted in blinded fashion to determine the number of CD3^+^IL-17A^+^, CD4^+^IL-17A^+^, and CD11b^+^ CCL20^+^ cells per high-power field (magnification 400×); cells were photographed with a microscope equipped with digital camera (BX63, Olympus, Tokyo, Japan).

### 2.4. Quantitative Evaluation of Cytokines, Chemokines and Related Cell Signaling Molecules by Real-Time PCR

Transcripts encoding cytokines, chemokines, and signaling molecules were evaluated in total RNA extracted from renal tissue from adult mice with TRIzol reagent (Invitrogen). SuperScript First-Strand Synthesis System (Invitrogen) was used to generate cDNA. Real-time PCR (RT-PCR) was performed in duplicate using the SYBR Green PCR Master Mix (Thermo Fisher, Waltham, WA, USA) according to the manufacturer’s instructions, using two independent RNA preparations. The primers for detection of transcripts encoding IL-6, Janus kinase 2 (JAK2), and signal transducer and activator of transcription 3 (STAT3) were purchased from SABiosciences (QIAGEN) and primers for all other transcripts are listed in Table 1. Cycling conditions were as per instructions from the company. PCR was carried out using Real-time PCR Detection System (Applied Biosystems) with relative expression analysis determined by reference to the housekeeping gene GAPDH as per the program provided by Applied Biosystems. The relative expression of the target genes was calculated using the ΔΔCt method. For relative quantification, the expression of RT-PCR was 1 in the wild-type (*Fli-1^+/+^*) group [14].

### 2.5. Measurement of Serum Cytokine IL-6 and IL-17A

Immunoreactive IL-6 and IL-17A in sera from adult mice (4 months or older) were evaluated quantitatively by enzyme-linked immunosorbent assay (ELISA) (R&D Systems and Thermo Fisher, respectively) as per the manufacturer’s instructions.

### 2.6. Measurement of Serum Double-Strand DNA Levels and Urine Protein Levels

Serum double-strand (ds) DNA antibody levels in sera from adult mice were measured by ELISA as per the manufacturer’s instructions (MyBioSource, San Diego, CA, USA). Urinary protein levels in adult mice (4 to 6 months old) were evaluated by dipstick (Wako).

### 2.7. Statistics

Results are presented as means ± standard deviation (SD). Quantitative data were analyzed using Student’s t-test with Welch’s correction or Mann–Whitney test as appropriate. A *p*-value <0.05 was considered significant. Statistical analyses were conducted using Excel add-ins and Statcel 4 Software (OMS Publishing, Saitama, Japan).

## 3. Results

### 3.1. Serum Cytokine Levels in MRL/lpr Fli-1^+/−^ Mice

**Figure 1:**
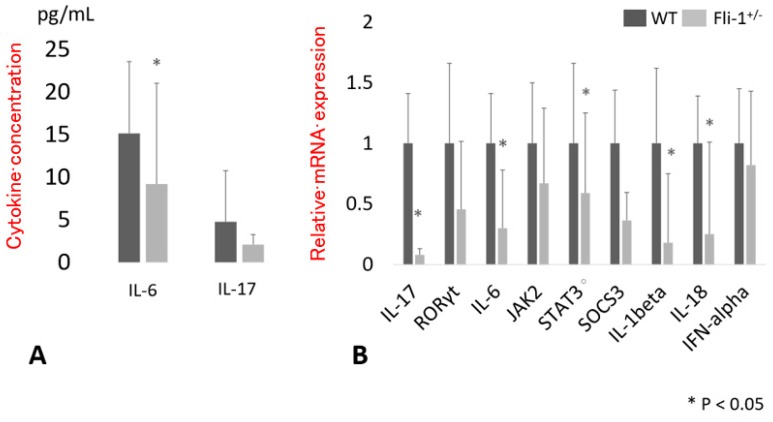
Serum cytokine levels and renal mRNA expression related to interleukin (IL)-6 and IL-17A cytokine signaling. (**A**) Serum IL-6 and IL-17A concentrations (pg/mL) in adult (4 to 10 months old) wild type (WT) (*n* = 11–15) and MRL/lpr *Fli-1^+/−^* mice (*n* = 15 per group). (**B**) Relative expression of cytokines and related signaling molecules in the kidney of adult (4 to 9 months old) MRL/lpr *Fli-1^+/−^* (*n* = 3–11) vs. WT mice (*n* = 4–13); * *p* < 0.05 is considered as significant.

Serum IL-17A concentrations were measured in samples from MRL/lpr *Fli-1^+/−^* mice and compared to those from WT MRL/lpr *Fli-1^+/+^* littermate controls. As shown in Figure 1A, IL-17A levels detected in sera from MRL/lpr *Fli-1^+/−^* mice were somewhat lower than those detected in littermate controls, although the difference did not reach statistical significance (*p* = 0.16). By contrast, and consistent with our earlier reports, levels of IL-6 in sera from MRL/lpr *Fli-1^+/−^* mice were significantly lower than those detected in littermate MRL/lpr *Fli-1^+/+^* controls (*p* < 0.05). Similarly, serum anti-ds DNA antibody levels and urine protein levels were significantly decreased in MRL/lpr *Fli-1^+/−^* mice (Figure S1A,B).

### 3.2. Expression of Transcript Encoding IL-17A in the Kidney of MRL/lpr Fli-1^+/^^−^ Mice

Messenger RNA was isolated from renal tissue from MRL/lpr *Fli-1^+/^^−^* mice and age-matched MRL/lpr *Fli-1^+/+^* littermate controls; expression of transcripts encoding IL-6, IL-17A, and downstream signaling molecules was evaluated by RT-PCR. As shown in Figure 1B, levels of IL-17A transcript were significantly lower in MRL/lpr *Fli-1^+/^^−^* mice compared to MRL/lpr *Fli-1^+/+^* littermate controls. Similar results were obtained in analyses of transcripts encoding IL-6 and the Th17-associated cytokines, IL-1β and IL-18, as well as the downstream signaling molecule, signal transducer and activator of transcription 3 (STAT3). By contrast, expression of transcripts encoding Janus kinase 2 (JAK2) and suppressor of cytokine signaling 3 (SOCS3) was somewhat diminished, although the results did not achieve statistical significance.

### 3.3. Reduced IL-17^+^ Cells in Renal Infiltrates from MRL/lpr Fli-1^+/^^−^ Mice

**Figure 2:**
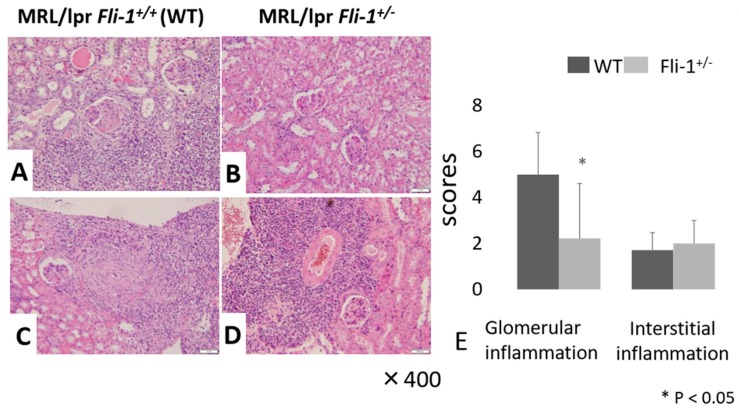
Renal pathology findings (Hematoxylin and Eosin (H&E) staining) in MRL/lpr *Fli-1^+/+^* (WT) and MRL/lpr *Fli-1^+/−^* mice. (**A**–**D**,**E**) H& E staining (**A**–**D**) and pathology scores (**E**) of glomerulus (Glom) and interstitial infiltrates (Int) in the kidney of age-matched, adult (4 months old or older) mice are shown (*n* = 7–9 per groups); * *p* < 0.05 is considered as significant.

**Figure 3:**
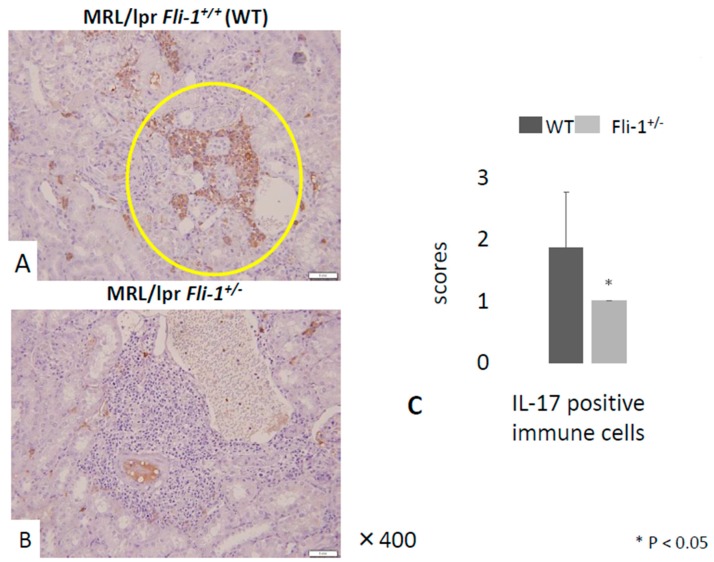
IL-17 staining of MRL/lpr *Fli-1^+/+^* (WT) and MRL/lpr *Fli-1^+/-^* mice. (**A**,**B**) Immunostaining of IL-17A in the kidney tissue from WT MRL/lpr and MRL/lpr *Fli-1^+/−^* mice (4 months old or older) and pathology scores in (**C**) (*n* = 7–9 per groups) are shown; * *p* < 0.05 is considered as significant.

Evaluation of renal tissue by H&E staining revealed a significant decrease in glomerular inflammation in MRL/lpr *Fli-1^+/^^−^* mice compared to their MRL/lpr *Fli-1^+/+^* counterparts. By contrast, we observed no differences in leukocyte infiltration to inflamed tubulointerstitial lesions and in part, perivascular lesions into the kidney (Figure 2A–E); whereas immunostaining with anti-IL-17A revealed a significant decrease of IL-17A^+^ cells in renal tubulointerstitial lesions in MRL/lpr *Fli-1^+/^^−^* mice compared to littermate controls (Figure 3A–C). Few to no cells were detected with anti-IL-17A within the glomerular lesions (Figure S1C,D).

### 3.4. Reduced Numbers of CD3^+^ IL-17^+^ and CD4^+^ IL-17^+^ Cells in the Renal Interstitium of MRL/lpr Fli-1^+/−^ Mice

**Figure 4:**
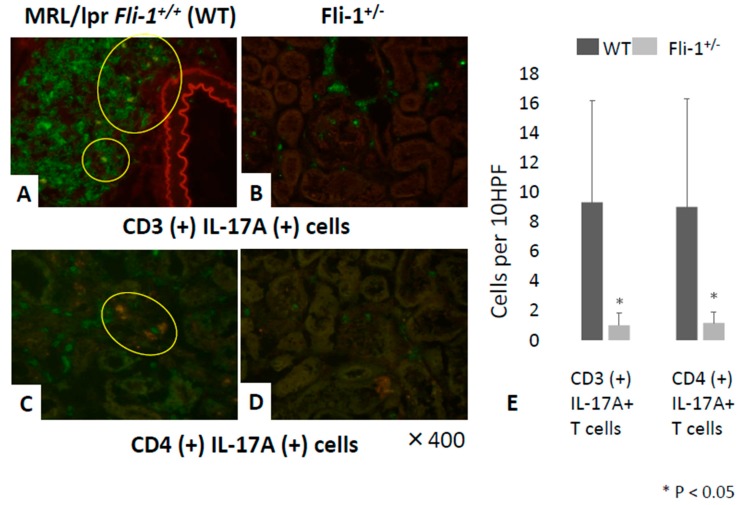
Detection of CD3^+^IL-17A^+^ and CD4^+^ IL-17A^+^ immune cells in the kidney of MRL/lpr *Fli-1^+/+^* (WT) and MRL/lpr *Fli-1^+/−^* mice using FITC-conjugated anti-CD3, anti-CD4, and Alexa Fluor 647-labeled IL-17 antibodies. Detection of CD3^+^IL-17A^+^ and CD4^+^ IL-17A^+^ cells in renal tissue from WT MRL/lpr and MRL/lpr *Fli-1^+/−^* mice (4 months old or older) is shown (**A**–**D**). Cell counts of CD3^+^ IL-17^+^ and CD4^+^ IL-17^+^ leukocytes and total counts from ten random high-power fields (HPFs) (*n* = 6–10 per groups) are shown (**E**); * *p* < 0.05 is considered as significant.

**Figure 5:**
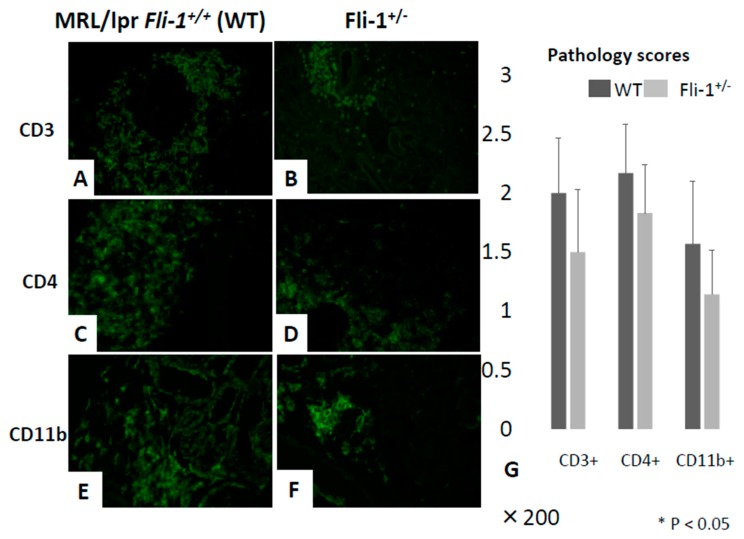
Relative infiltration of CD3^+^, CD4^+^, and CD11b^+^ immune cells in the kidney of MRL/lpr *Fli-1^+/+^* (WT) and MRL/lpr *Fli-1^+/−^* mice using FITC-conjugated anti-CD3, CD4, and CD11b antibodies. The grade of CD3^+^ (**A**,**B**), CD4^+^ (**C**,**D**), and CD11b^+^ (**E**,**F**) immune cell infiltration from WT MRL/lpr and MRL/lpr *Fli-1^+/−^* mice is shown (*n* = 6–10 per groups). Pathology scores of each group are shown (**G**); * *p* < 0.05 is considered as significant.

Immunofluorescence staining was performed to detect CD3^+^IL-17^+^ and CD4^+^IL-17^+^ cells in renal infiltrates of MRL/lpr *Fli-1^+/−^* and MRL/lpr *Fli-1^+/+^* control mice. As shown in Figure 4A–E, significantly fewer CD3^+^ IL-17^+^ cells (i.e., IL-17^+^ T lymphocytes) as well as CD4^+^IL-17^+^ cells (Th17 cells) were detected in renal tissue from MRL/lpr mice *Fli-1^+/−^* compared to littermate controls. By contrast, the renal tissues received similar scores (Figure 5A–D,G).

### 3.5. Detection of CCL20^+^CD11b^+^ Cells in Renal Tissue from MRL/lpr Fli-1^+/−^ Mice

**Figure 6:**
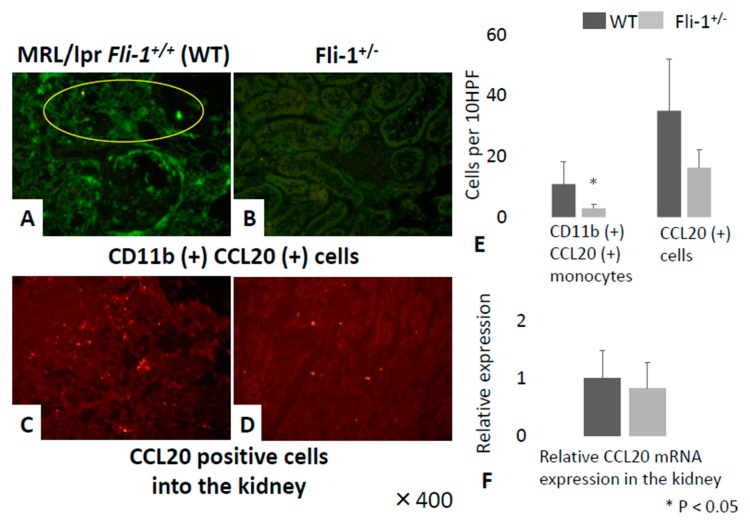
Detection of CD11b^+^ CCL20^+^ immune cells and infiltration of CCL20 positive cells in the kidney of MRL/lpr *Fli-1^+/+^* (WT) and MRL/lpr *Fli-1^+/−^* mice using FITC-conjugated anti-CD11b and Alexa Fluor 647-labeled anti-CCL20 antibodies. Detection of CD11b^+^ CCL20^+^ immune cells (**A**,**B**) and infiltration of CCL20 positive cells (**C**,**D**) in the kidney of both groups are shown; total counts from ten random high-power fields (HPFs) (**E**) (*n* = 6–7 per groups). Relative expression of transcript encoding CCL20 in the two groups (*n* = 3–4 per groups) (**F**) is shown; *p < 0.05 is considered as significant.

CCL20 is a major chemoattractant for Th17 cells and promotes infiltration into the kidney tissue [26]. As shown in Figure 6A,B,E, we observed a significant reduction in the numbers of CD11b^+^CCL20^+^ monocytes in renal tissue from MRL/lpr *Fli-1^+/−^* mice, in spite of similar infiltration of CD11b+ cells in the kidney of both groups (Figure 5E,F). Immunoreactive CCL20 was detected mainly in association with leukocytes infiltrating the kidney (Figure 6C,D). There were fewer CCL20^+^ cells in the renal tissue from MRL/lpr *Fli-1^+/−^* mice, but the difference was not statistically significant; expression of transcript encoding CCL20 reached similar levels in both strains of mice (Figure 6F).

## 4. Discussion

We observed fewer IL-17A^+^ lymphocytes and fewer CD11b^+^CCL20^+^ cells in the renal tissue of MRL/lpr *Fli-1^+/−^* mice compared to control MRL/lpr *Fli-1^+/+^* littermates. Expression of transcripts encoding IL-17A, related cytokines IL-6, IL-1β, IL-18, and the signaling molecule STAT3 was similarly reduced. The relationship between IL-17A levels and absence of a single Fli-1 allele was not immediately apparent. Among the possibilities, there may be an indirect effect of Fli-1 in promoting the development of lupus nephritis. In an earlier report, STAT3 was localized at the promoter region of the gene encoding IL-17A and served to regulate production of this cytokine in T lymphocytes [27]. In our previous study we found that Fli-1 directly regulated IL-6 expression and promoted the progression of lupus nephritis [13]. Furthermore, Hodge et al. described that IL-6 induces expression of Fli-1 via STAT3 [28]. It is conceivable that the reduction in IL-6 may have a direct impact on STAT3 and ultimately on the levels of IL-17A in renal tissue. Another possibility is that Fli-1 may regulate the expression and production of IL-17A in a more direct manner. IL-17A is produced by T lymphocytes; reduced numbers of these cells in renal infiltrates will also have an impact on levels of IL-17A detected. At this time, we are actively investigating the possibility that Fli-1 binds directly to the promoter of the gene encoding IL-17A.

We are also interested in understanding why fewer IL-17A^+^ cells were detected in renal tissue from MRL/lpr *Fli-1^+/−^* mice, despite similar levels of interstitial leukocyte infiltration when compared to MRL/lpr *Fli-1^+/+^* controls. Findings in earlier reports indicated that expression of chemokines was relatively lower in MRL/lpr *Fli-1^+/−^* mice [14]; this may result in an overall diminished degree of T cell chemotaxis and recruitment to the kidney. In fact, Sundararaj et al. reported that FLi-1 impacts MRL/lpr lupus nephritis through direct regulation of chemokine (C-X-C) motif receptor 3 (CXCR3) to reduce T cell activation, migration, and downregulation of CXCR3 ligands, *Cxcl9* and *Cxcl10* in the kidney [29]. CXCR3 plays an important role in mouse and human lupus, by regulating infiltration of Th1 and Th17 cells into the kidney [30,31]. Indeed, Steinmetz et al. reported that CXCR3 deficiency leads to significant morphological and functional improvement of lupus nephritis by interfering with immune cell trafficking of both Th1 and Th17 cells [31]. Therefore, it is possible that decreased CXCR3^+^ T cells by reduction of Fli-1 expression may result in lower Th17 cell infiltration into the kidney in MRL/lpr *Fli-1^+/−^* mice. Our results clearly demonstrate reduced numbers of CD11b^+^CCL20^+^ cells recruited to the kidneys of MRL/lpr *Fli-1^+/^^−^* despite similar levels of leukocyte infiltration and of CCL20 transcript. Since CCL20 is a ligand of CC chemokine receptor 6 (CCR6), which is predominantly expressed in Th17 cells, CCL20^+^ monocytes/macrophages induce migration of CCR6^+^ Th17 cells into the inflammatory cites [26,32,33]. Actually, Turner et al. reported that CCL20 expression was observed in mainly mononuclear cells of interstitial and periglomerular infiltrates (in contrast, podocytes and mesangial cells were almost negative) in murine experimental glomerulonephritis [26]. These observations may also indicate that reduction of CCL20^+^ monocytes affect local CCL20 expression and, in part, result in more decreased CCR6^+^ Th17 cells into the kidney of nephritic mice. Further investigation is needed to clarify the association of Fli-1 in CCL20 expression and infiltration of CCR6^+^ Th17 cells in lupus nephritis. In contrast to the findings presented here, several groups reported that IL-17A and the Th17 immune response have a relatively minor impact on the pathogenesis of lupus nephritis. For example, Schmidt et al. found that the degree of leukocyte infiltration in the kidneys of IL-17A-deficient MRL/lpr mice could not be distinguished from that detected in their IL17A-sufficient counterparts [34]. Likewise, neutralization of IL-17A by anti-IL-17A antibodies had no significant impact on the extent of nephritis in New Zealand Black/New Zealand White (NZB/NZW) F1 lupus-prone mice. Another report described that the urinary expression of Th17-related genes is increased in human SLE patients, however, the degree of upregulation is inversely related to systemic and renal lupus activity [35]. Interestingly, our results, which point to a critical role for IL-17A via the actions of Fli-1, are more consistent with those from previous clinical investigations [20,21,22]. Future studies from our group and others will clarify distinct mechanisms of renal inflammation promoted by IL-17A, the Th17 immune response, and their regulation by Fli-1.

## Figures and Tables

**Table 1:** Primers used for real-time polymerase chain reaction.

Primer Name	Forward Primer	Reverse Primer
IL-17	5′ACTTTCAGGGTCGAGAAGA-3′	5′TTCTGAATCTGCCTCTGAAT-3′
RORγt	5′AGCTTTGTGCAGATCTAAGG-3′	5′TGTCCTCCTCAGTAGGGTAG-3′
SOCS3	5′ACCTTCAGCTCCAAAAGCGAGTAC-3′	5′CGCTCCAGTAGAATCCGCTCTC-3′
IL-1β	5′CTTCAGGCAGGCAGTATCACTCAT-3′	5′TCTAATGGGAACGTCACACACCAG-3′
IL-18	5′GCTGTGACCCTCTCTGTGAA-3′	5′GGCAAGCAAGAAAGTGTCCT-3′
IFN-α	5′CATTCTGCAATGACCTCCAC-3′	5′TCAGGGGAAATTCCTGCAC-3′
CCL20	5′ATGGCCTGCGGTGGCAAGCGTCTG-3′	5′TAGGCTGAGGAGGTTCACAGCCCT-3′

## Supplementary Materials

The following are available online at https://www.mdpi.com/2073-4409/9/3/714/s1. Figure S1: Serum dsDNA antibody levels (A), urine protein levels (B) and representative finding of glomerular lesions by IL-17 staining in WT and *Fli-1^+/−^* MRL/lpr mouse.

## Abbreviations

CCL20	CC chemokine ligand 20
CCR6	CC chemokine receptor 6
CXCL	chemokine (C-X-C) motif ligand
CXCR 3	chemokine (C-X-C) motif receptor 3
Fli-1	Friend leukaemia integration 1
G-CSF	granulocyte colony stimulating factor
IFN	interferon
IL	interleukin
JAK	Janus kinase
MCP-1	monocyte chemoattractant protein-1
RANTES	Regulated on Activation, Normal T cell Expressed and Secreted
RORγt	Retinoic Acid Related Orphan Nuclear Receptor gamma T
SLE	systemic lupus erythematosus
SOCS	suppressor of cytokine signaling
STAT	signal transducer and activator of transcription
Th 17	T-helper 17
