# Supplementary material for: Ets Family Transcription Factor Fli-1 Promotes Leukocyte Recruitment and Production of IL-17A in the MRL/Lpr Mouse Model of Lupus Nephritis
Source: Cells. 2020 Mar 14;9(3):714. doi: 10.3390/cells9030714 (PMC7140643; doi:10.3390/cells9030714)

Supplementary figure 1

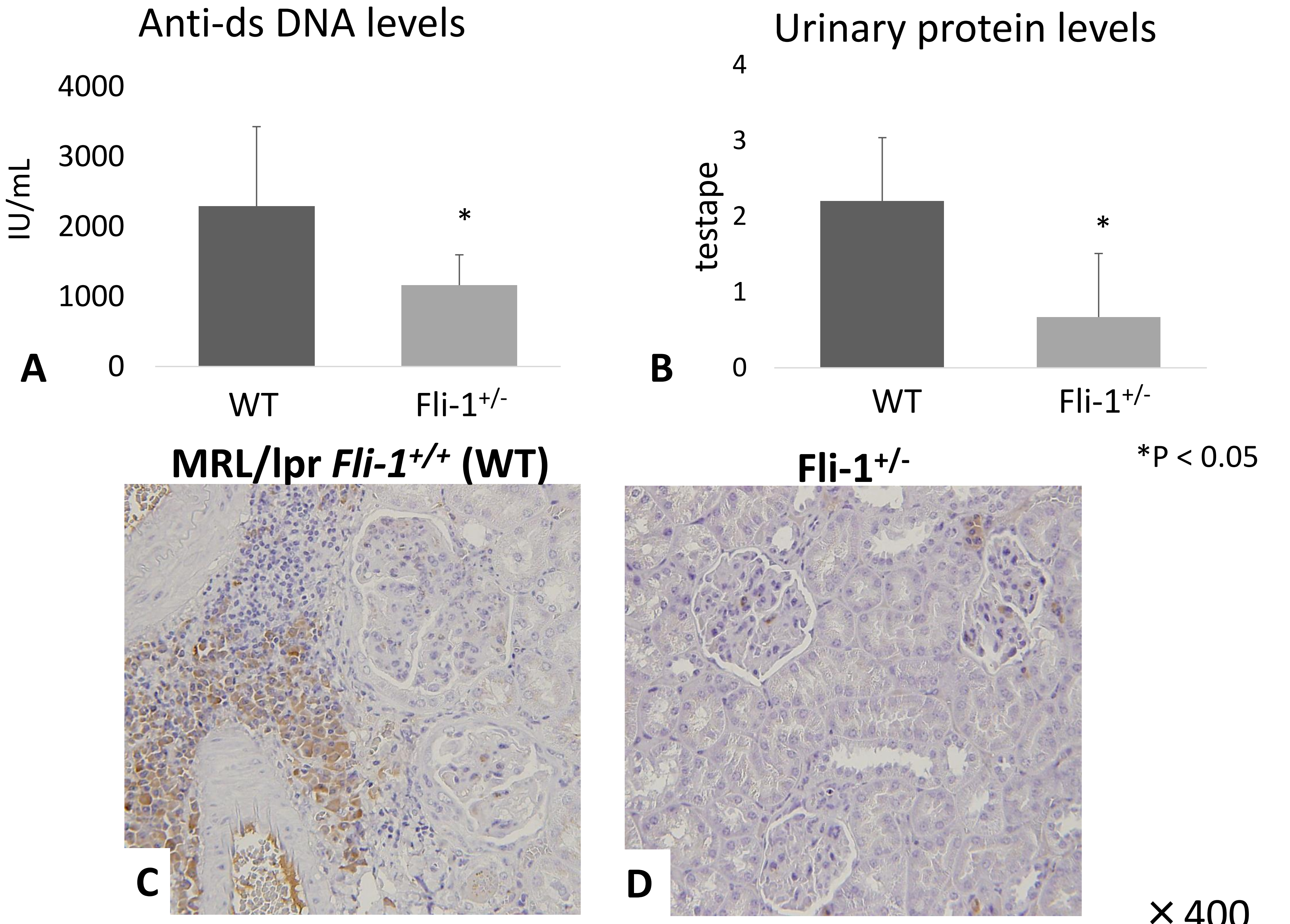

Supplement: Supplementary file 1 [file cells-09-00714-s001.pdf]
